# Annatto (*Bixa orellana*)-Based Nanostructures for Biomedical Applications—A Systematic Review

**DOI:** 10.3390/pharmaceutics16101275

**Published:** 2024-09-29

**Authors:** Vitória Regina Pereira da Silva, Natália Ornelas Martins, Carolina Ramos dos Santos, Elysa Beatriz de Oliveira Damas, Paula Lauane Araujo, Gabriella de Oliveira Silva, Graziella Anselmo Joanitti, Marcella Lemos Brettas Carneiro

**Affiliations:** 1 Laboratory of Bioactive Compounds and Nanobiotechnology (LCBNano), Campus Darcy Ribeiro, University of Brasilia, Brasilia 70910-900, Brazil; vitoriarpsilva@gmail.com (V.R.P.d.S.); nataliaornelas8@gmail.com (N.O.M.); crsbiotec@hotmail.com (C.R.d.S.); elysadamas@gmail.com (E.B.d.O.D.); paulaaraujoescs@gmail.com (P.L.A.); oliveiragaby101@gmail.com (G.d.O.S.); marbretas@gmail.com (M.L.B.C.); 2 Post-Graduate Program in Pharmaceuticals Sciences, Faculty of Health Sciences, Campus Darcy Ribeiro, University of Brasilia, Brasilia 70910-900, Brazil; 3 Post-Graduate Program in Biomedical Engineering (PPGEB), Faculty of Gama, University of Brasilia, Special Area of Industry Projection A, Brasilia 72444-240, Brazil; 4 Post-Graduate Program in Nanoscience and Nanobiotechnology, Institute of Biological Sciences, Campus Darcy Ribeiro, University of Brasilia, Brasilia 70910-900, Brazil

**Keywords:** *Bixa orellana*, annatto, nanotechnology, biomedical application, extract, oil, toxicity

## Abstract

Plants are a source of valuable organic chemical compounds with complex structures rich in therapeutic activities. The encapsulation of compounds in nanostructured systems is an alternative to avoid limitations, such as instability and low solubility, and to promote therapeutic use. The objective of the present review was to summarize the data in the literature on the physicochemical characteristics, biomedical efficacy, and toxicity of nanostructures containing extracts and oils obtained from annatto (*Bixa orellana*). For this, searches were conducted in the CINAHL, LILACS, Embase, FSTA, MEDLINE, ProQuest, PubMed, ScienceDirect, Scopus, and Web of Science databases. Studies that carried out the development, physical-chemical characterization, and evaluation of therapeutic efficacy and/or in vitro, in vivo, or clinical toxicity of nanostructures containing extracts and oils derived from annatto were included in the review. Of the 708 articles found, nine met the inclusion criteria. The included studies developed different nanostructures (nanofibers, nanocochleates, chitosan, lipid, polymeric, and metallic nanoparticles). These nanostructures showed leishmanicidal, photoprotective, antioxidant, antimicrobial, and immunomodulatory efficacy, and tissue regeneration potential with no or low toxic effects in the tested models. Thus, the present work supports the nanostructuring of annatto extracts and oils as a relevant approach to the development of new technologies for biomedical applications.

## 1. Introduction

Natural products are widely used in the health field to prevent, relieve symptoms, and cure diseases. These products are obtained from medicinal plants rich in bioactive molecules that present biological activities of interest, such as antitumor, antimicrobial, antioxidant, analgesic, and wound healing potential. However, the use of bioactive compounds has some limitations, such as low stability, limited absorption, hydrophobicity, low bioavailability, and others [1].

Nanotechnology presents several tools that can be used in the pharmaceutical field as an ally for the development of new therapeutic systems [1]. Nanotechnology uses a variety of nanomaterials with different characteristics, such as size, shape, and composition, which at the nanometric scale (10^−9^ m) enables several applications such as cosmetics, food, and pharmaceutical use [1]. Nanoencapsulation of bioactive compounds can solve solubility and stability issues, as well as shield the bioavailability and unique properties of bioactive compounds present in plants [2]. 

*Bixa orellana* is a native tree from Brazil popularly known as annatto. The fruit is red, globular, and has ovoid capsules from which annatto oil and annatto extract can be obtained. It is commonly used as a natural dye, and it has been used as an insect repellent and sunscreen by traditional communities [3]. Several pharmacological studies have highlighted *Bixa orellana*’s therapeutic effects, encompassing photoprotective, antioxidant, antidiabetic, anti-inflammatory, antimicrobial, anticancer, and anthelmintic properties [4,5,6,7].

The use of annatto extracts and oil associated with nanostructures is observed both with the complete extract and the isolated bixin [2]. Due to its hydrophobic characteristic, the main nanostructures reported for association with annatto are solid lipid nanoparticles and nanoemulsions that allow its encapsulation inside the nanoparticle, promoting better dispersion and stability [8,9]. In addition to the encapsulation of oil/extract, the association of annatto can be incorporated in the green synthesis of metallic nanoparticles, for antimicrobial and antioxidant applications [10,11].

The present systematic review performed a study to evaluate the characteristics of the nanostructures in which the annatto oil or extract can be encapsulated, as well as its main biological applications and toxicity.

## 2. Methodology

### 2.1. Protocol and Registration

The present study was conducted in accordance with the Preferred Reporting Items for Systematic Reviews and Meta-Analyses (PRISMA) guidelines [12]. The protocol of this systematic review was registered in the International Prospective Register of Systematic Reviews [13] with registration number: CRD42023448167.

### 2.2. Eligibility Criteria

#### 2.2.1. Inclusion Criteria

This systematic review used as inclusion criteria studies that performed nanostructuring of *Bixa orellana* oil or extract and evaluated biological activities or toxicological profile. 

#### 2.2.2. Exclusion Criteria

Studies were excluded for the following reasons: (I) Studies of non-nanostructured *Bixa orellana*. (II) Studies that performed nanostructuring of compounds isolated from *Bixa orellana*. (III) Studies that did not evaluate the parameters of morphology, hydrodynamic diameter, zeta potential, or polydispersion index of nanostructures. (IV) Studies that did not evaluate biological activity or toxicological profile of *Bixa orellana* nanostructures. (V) Review articles, book chapters, theses, letters, conference abstracts, and patents. 

### 2.3. Information Sources and Search Strategy

Individual search strategies were designed for each of the following bibliographic databases: CINAHL, LILACS, Embase, FSTA, MEDLINE, ProQuest, PubMed, ScienceDirect, Scopus, and Web of Science (Supplementary Table S1). The search in the databases was conducted on 5 and 6 June 2023. No year of publication or language filter restrictions were applied. Duplicate references were eliminated using the reference management web application Rayyan^®^ [14].

### 2.4. Study Selection

The articles were selected in two phases: screening of titles and abstracts (phase 1) and reading of full text (phase 2). For both phases, three pairs of authors (VS and NO; ED and PA; CR and GS) were formed to independently analyze the studies. In phase 1, the pairs reviewed the titles and abstracts of all references identified in the electronic databases and selected the articles that appeared to meet the inclusion criteria, using the systematic review web application (Rayyan^®^) [14]. In phase 2, the pairs analyzed the full text of the articles selected in phase 1 and excluded studies that did not meet the inclusion criteria (Supplementary Table S2). A third author was consulted if disagreements between the two initial reviewers were not resolved by consensus. With the selected articles, the pairs independently extracted the relevant data on materials and methods (plant part and extraction method used, extract characterization, nanoparticle type and production, storage conditions and stability analysis, model and evaluation of biological activity and/or toxicity, treatment regimen) and results (result of extract characterization, nanoparticle characterization, stability analysis, evaluation of biological activity and/or toxicity). 

### 2.5. Risks of Bias and Quality in Individual Studies

The quality of the included articles was assessed by applying a 19-question form based on and adapted from the ARRIVE Guidelines (Supplementary Table S3) related to methodology and outcome measurement [15]. The questionnaire comprised 19 question options: YES for high study quality, NO for low study quality, UNCLEAR indicated that the information is present in the study but not entirely clear, NOT AVAILABLE when the information was absent, and NOT APPLICABLE when the question did not apply to the analyzed study. These questions were identified by the reviewers and used to classify the studies based on the proportion of YES responses according to the established criteria. Based on this assessment, the studies were categorized as high quality when they exhibited a rate above 70%, moderate quality in the 50% to 69% range, and low quality when they recorded a rate below 49%. A second questionnaire was administered for studies that underwent in vivo analysis using the 10-question SYRCLE RoB Toll to assess the risk of selection, performance, detection, attrition, and other biases (Supplementary Table S4) [16]. YES responses indicated a low risk of bias, NO indicated a high risk of bias, and UNCLEAR indicated that bias could not be attributed. The items were answered for each study by two reviewers individually, and disagreements were resolved by a third reviewer. 

## 3. Results

### 3.1. Study Selection

**Figure 1:**
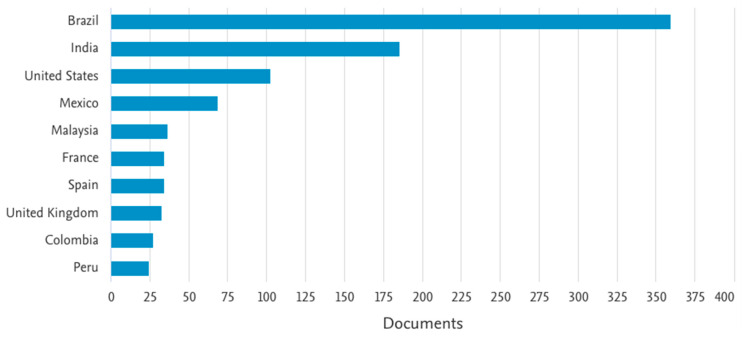
Number of publications listed in Scopus database by regions or countries. Data were obtained on 6 June 2023.

A search conducted in the Scopus database on 6 June 2023, using the search string “(bixa OR bixaceae OR ‘bixa orellana’)” identified a total of 4271 documents. This substantial number of publications highlights the interest and relevance of topics related to *Bixa orellana* within the scientific community. Among these documents, the following regions/countries with the highest academic production stand out: Brazil > India > United States > China > United Kingdom > Mexico > Malaysia > Egypt > Spain > France. Notably, Brazil leads the production with 1018 publications, representing 23.83% of the total, followed by India with 17.91% of the contributions. This can be observed in Figure 1.

By applying a filter to the results using the search string “(bixa OR bixaceae OR “*bixa orellana*”) AND (“nano-material” OR “nano-scale material” OR “nano-scale structure” OR “nano-sized material” OR “nano-sized structure” OR “nano-structure” OR “nano-structured material” OR “nanoscale material” OR “nanoscale structure” OR “nanosized material” OR “nanosized structure” OR nanostructure OR “nanostructured material” OR nanostructures OR nanomaterial)”, to identify specific studies that addressed the relationship between annatto and NPs (nanoparticles), a total of 186 documents were retrieved. According to data from the Scopus database, the countries/regions that stood out in publications on the combination “bixaceae AND nanomaterial” were India (69 publications), Brazil (27 publications), and China (18 publications), among others.

**Figure 2:**
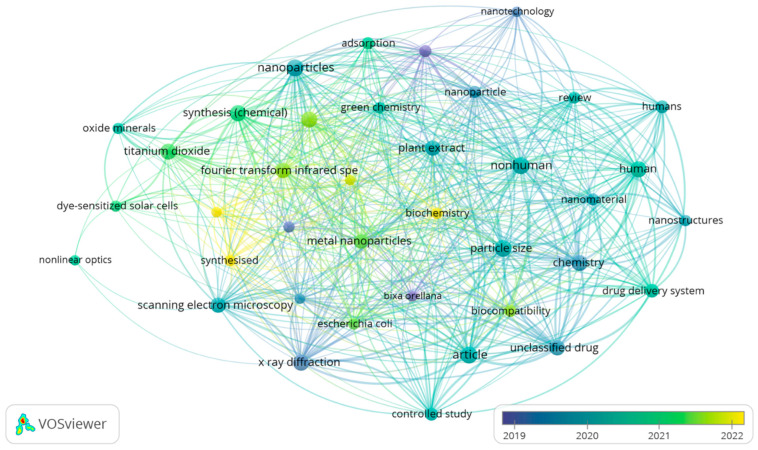
Bibliometric study of publications listed in the Scopus database, with MeSH terms for *bixaceae* and nanomaterials, using the VOSviewer program (version 1.6.18), with at least five co-occurrences and full count configurations. Data were obtained on 8 August 2023.

By performing a bibliometric analysis of the results from the most recent search in the Scopus database, using VOSviewer 1.6.18 software, it was possible to identify 93 relevant terms. This survey was carried out considering the co-occurrence of at least five terms using “full count” settings. Figure 2 illustrates the interconnection of three distinct clusters among the most frequently used terms. Among these terms, the prominent interest of the scientific community in the topic evaluated by concepts such as plant extract, particle size, and nanoparticles stands out.

**Figure 3:**
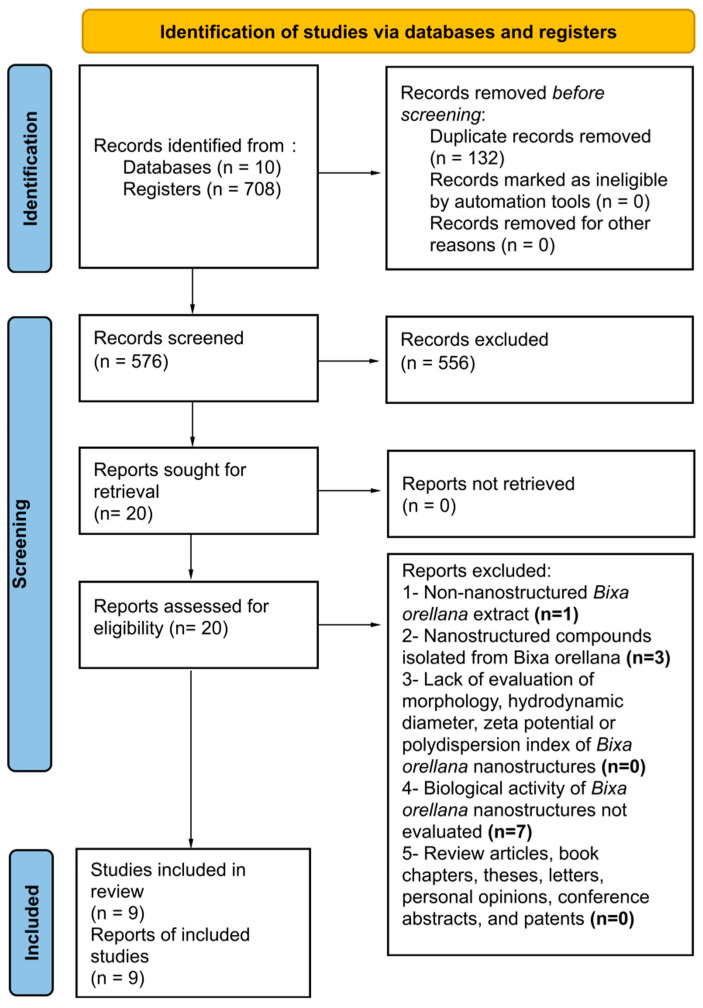
PRISMA flow diagram.

The data in Figure 1 and Figure 2 were acquired exclusively from the Scopus database to provide a comprehensive overview of the topics covered in this context. However, within this systematic review, a total of 708 studies were identified from different databases (24 from PubMed, 35 from Medline, 2 from LILACS, 8 from FSTA, 10 from CINAHL, 44 from Scopus, 57 from Web of Science, 143 from Science Direct, 20 from Embase and 365 from ProQuest) (Supplementary Table S1). After removing duplicates, 576 studies remained, and an assessment of ‘titles and abstracts’ resulted in the exclusion of 556 studies. The remaining 20 articles were subjected to a full-text analysis. This procedure resulted in the exclusion of 11 articles according to the exclusion criteria (Supplementary Table S2). In the end, 9 articles were retained and incorporated into this systematic review. A detailed flowchart of this process is displayed in Figure 3.

### 3.2. Characteristics of the Included Studies

All the included studies were research articles that explored the promising advantages with the association of nanostructures and annatto extract/oil. These studies were conducted in various countries: Brazil (*n* = 5), India (*n* = 1), Greece (*n* = 1), Cuba (*n* = 1), and Bangladesh (*n* = 1), with publication dates ranging from 2017 to 2023 and all of them being published in English. The main characteristics of these studies are summarized in Table 1. 

The types of nanoparticles (NPs) utilized encompassed lipid nanoparticles (*n* = 3), nanofibers (*n* = 2), polymeric nanoparticles (*n* = 2), and metallic nanoparticles (*n* = 2). These nanoparticles were primarily characterized by their particle size (*n* = 5), zeta potential (*n* = 5), and polydispersity index (PdI) (*n* = 4) using dynamic light scattering and electrophoretic mobility. Additionally, several studies examined NP absorbance or undertook physicochemical characterization through UV-vis spectrophotometry. Furthermore, some studies also assessed encapsulation efficiency, pH, and morphology. 

Six (*n* = 6) studies used *Bixa orellana* seed extract, with three (*n* = 3) using the maceration method with acetone, two (*n* = 2) using maceration with ethanol, and another two studies (*n* = 2) using water extraction. Two other studies used *Bixa orellana* oil and seed essential oil but did not specify the extraction methods.

Among all the included studies, all of them assessed biological activity in in vivo models, while only three (*n* = 3) evaluated both in vitro and in vivo activity, and one (*n* = 1) study assessed activity in an ex vivo model and human volunteers. In vitro studies evaluated cytotoxicity/proliferation (*n* = 6), photoprotective efficacy (*n* = 2), cell morphology and differentiation (*n* = 1), antileishmanial assay (*n* = 1), anti-amastigote (*n* = 1), and antimicrobial activity (*n* = 1). Animal models were considered adequate, and ethical committee approval was reported in all studies that utilized them.

### 3.3. Quality of Individual Studies

When evaluated according to the adapted ARRIVE Guidelines, as shown in Table 2, all included articles (*n* = 9) were classified as high quality. The majority of the articles clearly reported the source of annatto extract (*n* = 8), however, four did not present the extraction methodology applied (*n* = 4). Characterization of the annatto extract was conducted in five studies (*n* = 5). Among the selected articles, eight (*n* = 8) provided clear descriptions of the procedural steps in the experiments, seven studies (*n* = 7) detailed all characteristics of the animals and cells used, and some articles explicitly mentioned the presence of control groups (*n* = 7), relevant biological variables for the outcomes (*n* = 8), and indicated the absence of conflicts of interest (*n* = 9).

The assessment of bias risk based on the SYRCLE RoB Toll guidelines, applied to studies that presented in vivo activity, is summarized in Table 3. The evaluated studies did not clearly describe information regarding allocation, randomization, and blinding. The studies did not make it clear whether the animals were randomly housed and if the animals were randomly selected for outcome assessment. Two studies clearly indicated that the experimental groups were similar at the study’s outset. Only one study made it clear that allocations were adequately concealed, while two studies did not adequately address incomplete outcome data. Two of the studies were considered free from other issues that could result in a high risk of bias, and all the assessed studies were free from selective outcome reporting.

### 3.4. Synthesis of Results

The studies employed distinct types of extracts derived from *Bixa orellana*, with the majority utilizing the species’ oil (*n* = 4) and aqueous extract (*n* = 4), while solely one instance involved the employment of the powdered form of the species (*n* = 1). From this set of studies, three investigations presented a minimum of one extract characterization analysis, while two studies introduced a plurality of assays (*n* = 3). Four studies did not provide data regarding the characterization of the extract used to develop the nanoparticles (*n* = 4).

The research strategies for *Bixa orellana* nanostructures encompassed its application within various nanostructure types, including nanofibers (*n* = 2), solid-lipid nanoparticles (*n* = 1), nanostructured lipid carriers (*n* = 1), polymeric nanodispersions (*n* = 1), metallic nanoparticles (*n* = 2), chitosan nanoparticles (*n* = 1), and nanocochleates (*n* = 1). All nanostructures were minimally characterized in terms of size, measured in nanometers. Within the scope of the studies, the prevailing number of nanostructures exhibited dimensions below 200 nm (*n* = 5), whereas in the other cases, the sizes extended up to 500 nm. 

The nanostructures were subjected to various biomedical applications analysis, including photoprotective effects (*n* = 2), effects against leishmaniasis (*n* = 2), and anti-inflammatory properties (*n* = 1). Two studies assessed both antimicrobial and anticancer effects within the same investigation. The assays regarding therapeutic effects encompassed diverse strategies, ranging from in vitro studies involving cells and/or animal investigations, with one conducted in rats and another in mice. Only one study evaluated the biological effect on human skin tissue. Solely one study evaluated the properties of the nanostructures to produce scaffolds for cultivated meat.

## 4. Discussion

To the best of our knowledge, this work is the first review addressing nanostructures containing raw annatto products (extracts and oils). The rich chemical matrix of natural products found in annatto can potentially enhance their biomedical effects due to the synergy among the various compounds [22,23]. Additionally, the use of raw materials derived from annatto can reduce the costs associated with the compound isolation process, which poses a challenge that may limit the large-scale production of biomedical products containing isolated compounds [24]. Therefore, this systematic review summarizes the literature on the physicochemical characteristics, biomedical efficacy, and toxicity of nanostructures containing extracts and oils obtained from annatto, with the aim of guiding further scientific investigations using these products.

The studies included in this review highlight the development of solid lipid nanoparticles, nanostructured lipid carriers, nanocochleates, polymeric nanodispersions, chitosan nanoparticles, nanofibers, and metallic nanoparticles (zinc and silver), using fixed and essential oils and aqueous and dry extracts from different annatto parts, such as bark, seeds, and leaves. Due to the heterogeneity of the nature of the nanostructures and annatto products used, this work is an attempt to provide a comprehensive view of the impacts of annatto products on the physicochemical characteristics of the nanostructures produced as well as the biological effectiveness of these nanomaterials.

### 4.1. Influence of Annatto Products on the Physicochemical Characteristics of Nanostructures

As described in Table 1, lipid nanostructures were the most used platform to nanostructure annatto products (33.3%; 3 of 9 studies included), followed by nanofibers (22.2%; 2 of 9 studies included), metallic (22.2%; 2 of 9 studies included), and polymeric (22.2%; 2 of 9 studies included). These nanostructures present very different physicochemical characteristics when compared.

Regarding size, lipid nanoparticles produced using fixed and essential annatto oils had sizes ranging from 52 to 200 nm [18,19]. The literature describes the size of these nanostructures as varying between 50 and 1000 nm [25]. Interestingly, in the study by Andréo-Filho (2017) [3], solid nanoparticles containing annatto oil ranged in size from 0.20 to 1.42 µm. This disparity in sizes is associated with the synthesis methodology used. The solid lipid nanoparticles containing annatto oil and produced using a high-energy homogenization process showed significantly smaller particle sizes (~0.20 µm) compared to the same formulation subjected to a low-energy process through heating (~1.42 µm) [3]. High-energy methodologies, such as ultrasonication and high-pressure homogenization, are widely used because they significantly reduce the size of nanoparticles. These methodologies focus on shear forces, cavitation, and collision of particles necessary to break larger particles into smaller ones [3,18].

Cellulose acetate nanofibers containing annatto seed extract ranged in size from 269 to 420 nm [9,21]. In these nanostructures, the presence of the extract had a relevant effect on size determination. The biomolecules bixin and norbixin are capable of interacting with the functional groups of cellulose, acting as plasticizers. This effect significantly reduced the size of the nanofibers from 468 to 269 nm and also reduced the stiffness of the nanofiber mesh compared to the formulation without the extract [9]. 

Annatto polymeric nanoparticles ranged in size from 53 to 340 nm. The interaction of annatto products with the nanoparticle components can impact the size of the nanostructure formed, as observed in the study by Ntohogian (2018) [20], in which it was possible to observe an increase from 250 to 287–340 nm in the size of chitosan nanoparticles in the presence of annatto dry extract. 

Metallic nanoparticles are described as having particle sizes in the range of 10 to 100 nm. Among the studies included in the review, silver and zinc nanoparticles synthesized by green synthesis using annatto extract showed sizes ranging from 28.6 to 92.9 nm. In the study by Gharpure (2022) [11], the synthesis of zinc nanoparticles was carried out in a comparative way using extracts from the annatto seed husks, seeds, and leaves. These different extracts differ in their chemical composition, making it possible to observe that aromatic and vinylic compounds are present in aqueous extracts of the annatto leaf; the seeds contain phenolic and aromatic compounds, flavonoids, and fatty acids; phenolic compounds, steroids, flavonoids, carotenoids, fatty acids, and hydrocarbons are present in the seed husks [11]. 

These differences in chemical characterization were reflected in differences in the physicochemical characterization of the nanoparticles formed. Zinc nanoparticles synthesized with annatto seed extract presented an almond-like shape in the range of 220–440 nm, while nanoparticles synthesized from annatto leaves and seed husks presented a spherical shape in the range of 169–259 and 278–654 nm, respectively. Additionally, these nanoparticles also presented different surface areas, with the largest surface area observed in the nanoparticles synthesized using annatto leaf extract, followed by those synthesized with seed extract and the seed husks [11].

The zeta potential refers to the electrostatic charge of the nanoparticles’ surface. Systems that have a zeta potential of ± 30 mV are considered highly stable due to the repulsion effect between the charges [26]. Chitosan nanoparticles containing annatto extract showed a higher zeta potential value (47 mV) compared to the formulation without the extract (36 mV) [20]. Also, the presence of annatto oil in the formulation of nanostructured lipid carriers contributed to the stability of the system by increasing the modulus of the zeta potential [18]. Consequently, it was observed that nanocarriers containing annatto oil showed fewer variations in hydrodynamic size after 90 days of storage compared to nanocarriers without annatto oil [18]. In both formulations, not only the presence but also the concentration of annatto extract and oil determined changes in the zeta potential value, making it possible to observe that the increase in zeta potential was proportional to the increase in the concentration of annatto products [18,20].

The stability of a nanostructured system is also directly associated with the organization of its crystalline structure. In nanostructured lipid carriers, the interior of the nanoparticles presents a less organized crystalline structure compared to solid lipid nanoparticles. This low organization can increase the level of entrapment of the encapsulated bioactive compounds; on the other hand, it impairs the stability of the system [18]. The nanostructured lipid carriers containing annatto oil produced in the study by Ferreira et al. (2021) [18], showed greater fluidity and amorphous organization of the crystalline structure of their interior compared to carriers without the oil.

The development of nanostructures using annatto products is a tool capable of optimizing the interaction of annatto bioactive compounds with biological systems and enhancing their therapeutic activities [27]. Different biological applications were evaluated to determine the therapeutic efficacy and toxicity profile of the nanostructures containing annatto products. The results of these evaluations will be described below.

### 4.2. Therapeutic Efficacy Profile of the Nanostructures Containing Annatto Products

#### 4.2.1. Photoprotective and Healing Activity

Solar irradiation by ultraviolet rays is the cause of more than 50% of skin tumors in humans [28]. Sun protection products consist of chemical agents, such as titanium and zinc dioxide, which protect against UV radiation by absorbing or reflecting the rays [3]. The association of sunscreen formulations with natural products that have photoprotective and antioxidant activities is relevant for increasing photoprotection. The presence of pigments, such as bixin and norbixin, guarantees photoprotective and antioxidant activities to annatto products. Bixin has already been described as capable of activating nuclear factor-E2-related factor 2 (NRF2) and triggering a series of protective responses against solar radiation-induced damage and carcinogenesis through DNA detoxification and repair mechanisms [29].

Nanoparticles can be used to encapsulate natural products and promote increased sun protection. This effect is a result of the higher surface area ratio promoted by the reduced size of the nanoparticles, where greater surface coverage is achieved [3]. The photoprotection of formulations is determined by the sun protection factor (SPF) value. SPF values between 2 and 12 result in minimal photoprotective activity, whereas SPF values between 12 and 30 result in moderate protective activity, but high photoprotective activity is found in formulations that present SPF values greater than 30 [20]. Chitosan nanoparticles containing 20, 40, and 60% natural and ultrafiltered annatto extract showed minimal photoprotective effect ranging in SPF values from 2.34 to 2.76 [20]. When associated with chemical compounds used in the formulation of sunscreens, the photoprotective activity is enhanced, as observed with solid lipid nanoparticles containing annatto oil and the compound octyl methoxycinnamate, presenting SPF values between 19.7 and 21.7 [3].

Nanofibers are a type of nanostructure quite relevant for therapeutic use in dermal wounds. The random alignment of the fibers results in the construction of a mesh similar to the structure of the extracellular matrix. This structure facilitates cell adhesion and diffusion of oxygen and nutrients, thus promoting cell proliferation and accelerating healing processes [9,21,30]. In the study by Santos et al. (2021), cellulose acetate nanofibers containing annatto seed extract facilitated the adhesion of fibroblasts to their surface in vitro and showed an immunomodulatory effect in vivo with inhibition of the recruitment of immune system cells and no scarring evidence at the implantation site 15 days after the intervention [9].

#### 4.2.2. Leishmanicidal Activity

Leishmaniasis is an endemic disease of tropical regions of South and Central America, Asia, Africa, and Southern Europe. It is caused by infection with parasites of the genus *Leishmania*. Leishmaniasis is classified into visceral, cutaneous, mucosal, and post kala-azar dermal leishmaniasis. The infection begins after being bitten by mosquitoes of the *Lutzomyia* and *Phlebotomus* genus, in which the parasites in the promastigote form are transmitted to the human host, where they develop in the amastigote form, inside cells of the host’s phagocytic mononuclear system, mainly macrophages [31]. Treatments for leishmaniasis consist of meglumine antimoniate, sodium stibogluconate, amphotericin B, miltefosine, paromomycin, and pentamidine [31]. Despite being widely used, these drugs have high toxicity and high cost, and some are administered parenterally. These factors result in low adherence by patients [18].

Nanoparticles can direct antiparasitic molecules into organs rich in macrophages such as bone marrow, liver, lymph nodes, and kidneys, enhancing leishmanicidal activity. Furthermore, nanoparticles ranging in size from 50 to 500 nm are rapidly phagocytosed by macrophages, which may be interesting for the treatment of amastigotes of leishmania [32]. In the study by Ferreira et al. (2021) [18], nanostructured lipid carriers containing 2 and 4% annatto oil showed 70–90% leishmanicidal activity in reducing amastigote forms of *L. major*, being even significantly more effective than the standard drug meglumine antimoniate. Interestingly, free annatto oil did not have a leishmanicidal effect [18].

In the study by Machin et al. (2019) [19], nanocochleate formulations containing annatto essential oil showed lower in vitro leishmanicidal activity in amastigote forms of *L. amazonensis* compared to free annatto essential oil. Nevertheless, in an in vivo model, nanocochleates were able to reduce the size of lesions in animals infected with *L. amazonensis* to a greater extent compared to the free essential oil and in a similar way with animals treated with glucantime [19].

Nanocochleates are cylindrical lipid nanoparticles of the liposome family formed from the interaction between negative phospholipids and divalent cations [33]. The multilamellar characteristic of nanocochleates allows bioactive compounds to associate both externally and internally, being slowly released by desorption, dissolution, or diffusion [19]. The slow release of annatto essential oil may have impaired the in vitro leishmanicidal activity due to the short exposure time (48 h). In the in vivo test, the exposure time was longer, promoting the release of annatto essential oil and evidencing its leishmanicidal effectiveness [19].

#### 4.2.3. Antioxidant and Antimicrobial Activity

Microbial resistance to the effect of antimicrobial drugs is a worldwide problem and has made the treatment of infections an arduous process [34]. Metallic nanoparticles have been described in the literature as a potential antimicrobial agent [34]. These nanoparticles can reduce the viability of bacteria through the production of reactive oxygen species and promoting changes in the structure of the microbial cell membrane, culminating in the leakage of intracellular content. Furthermore, Zinc ions (Zn^2+^) inhibit cellular active transport, inactivate enzymes, and disrupt amino acid metabolism. In fungi, metallic nanoparticles are responsible for the uncontrolled accumulation of cellular components and interference with cellular functioning [11].

The different bioactive compounds of annatto extract can be used in the green synthesis of metallic nanoparticles. Compounds such as flavonoids, steroids, and phenolic compounds act as chelating agents and form complexes with metal ions, stabilizing them and forming nanoparticles [11]. In addition to their role in the formation of nanoparticles, the bioactive compounds in the extract may present synergism in biological activities.

Zinc nanoparticles produced using annatto leaf, seed, and bark extract were tested for antibacterial activity on strains of *Staphylococcus aureus*, *Bacillus subtilis*, *Escherichia coli*, and *Pseudomonas aeruginosa*. Although the crude extracts of these products did not show antibacterial activity, when associated with zinc nanoparticles, the formulation was able to reduce the viability of strains of *S. aureus*, *B. subtilis*, and *E. coli*. However, it was not successful in reducing the viability of *Penicillium* sp., *Aspergillus flavus*, *Fusarium oxysporum*, and *Rhizoctonia solani* fungal strains [11]. Silver nanoparticles produced with aqueous extract of annatto seeds were tested for antibacterial activity against strains of *S. aureus*, *E. coli*, *Shigella dysenteriae*, and *Shigella boydii* but showed antibacterial activity restricted to only strains of *S. dysenteriae* [10].

It was also possible to observed that the physical-chemical parameters of nanostructures modulate interactions with cells. The process of cellular absorption and targeting between intracellular compartments is influenced by the size of the nanoparticle and its charge [18,35]. Zinc nanoparticles produced with extracts from annatto leaves showed smaller particle sizes and greater antibacterial activity against strains of *S. aureus* and *B. subtilis*, demonstrating that perhaps smaller nanoparticles are being more internalized in bacterial cells [11].

Oxidative stress is characterized by a high concentration of free radicals that promote changes in the structure and function of nucleic acids, lipids, and proteins. These changes are associated with the pathogenesis of neurodegenerative and cardiovascular diseases, and cancer [36]. The establishment of the oxidative stress state occurs due to the inefficiency of endogenous antioxidant mechanisms in neutralizing free radicals. In this sense, silver nanoparticles play a relevant antioxidant role because they have unpaired electrons capable of binding to free radicals and stabilizing them. This antioxidant activity was observed in the study by Maitra et al. (2023) [10], in which silver nanoparticles synthesized using annatto extract were 78.86% effective in reducing the DPPH radical [10].

Additionally, the association of bioactive compounds from the extract used in green synthesis on the structure of metal nanoparticles is a phenomenon already described in the literature, as demonstrated on the FTIR results found by Sharma et al. (2024) [37]. In this sense, the result found in the study by Maitra et al. (2023) can also be associated with the annatto extract itself, which in the study by Quiroz et al. (2019) showed antioxidant activity in the ABTS (2,2′-azino-bis(3-ethylbenzothiazoline-6-sulfonic acid)), FRAP (Ferric-Reducing Antioxidant Power) and DPPH tests [38].

#### 4.2.4. Antitumoral Activity

Cancer is characterized by the occurrence of mutations associated with the activation of proto-oncogenes and the inactivation of tumor suppressor genes. These alterations lead to disordered proliferative rates in cells and other changes favoring tumor progression, such as immune system evasion, energy metabolism reprogramming, heightened angiogenesis, and the occurrence of metastases [39]. According to the Global Cancer Observatory, approximately 21.9 million new cases of cancer are expected to be reported worldwide by 2025 [40]. The shared characteristics between healthy and tumor cells, coupled with the high heterogeneity expressed in tumor cells, pose a significant challenge in cancer treatment.

Several studies have elucidated the potential of metallic nanoparticles in reducing the viability of tumor cells [41]. The dissolution of metals from nanoparticles can induce changes in mitochondrial functioning, DNA damage, increased intracellular concentrations of reactive oxygen species, and rupture of the cell membrane [42]. In a study conducted by Maitra (2023) [10], silver nanoparticles synthesized using the aqueous extract of annatto seed demonstrated the ability to inhibit the proliferation of MCF-7 breast cancer cells in a dose-dependent manner, exhibiting a maximum inhibition of 68.3% at a concentration of 31.25 µg/mL.

While zinc nanoparticles are described in the literature as potential antitumor agents, with reported efficacy in reducing the viability of HCT-116 colon cancer cells [43], a study by Gharpure (2022) [11] observed that zinc nanoparticles synthesized using extracts from the bark, seeds, and leaves of annatto did not exhibit antitumor activity in HCT-116 colon cancer cells.

### 4.3. Toxicity of Annatto Nanostructures

The widespread use of products obtained from annatto in the food industry and in traditional medicine reveals signs of their safety. Analyzing the composition of annatto extracts, it is possible to observe that bioactive compounds with high potential for toxicity, such as alkaloids, saponins, and glycosides, are present in low concentrations, while compounds with low potential for toxicity, such as terpenoids, flavonoids, and phenolics are present in high concentrations [44]. The clinical study by Zegarra et al. (2007) administered 250 mg of powdered extracts of annatto leaves to participants three times a day for 6 months and no harmful adverse effects were observed, evidencing the biocompatibility of these extracts [45].

One of the bases of nanotechnology is the principle of improvements of nanomaterials to present new physicochemical properties to the same materials structured on larger scales. These new properties may favor the biological activities of the compounds, but may also change their toxicological profile. Mechanisms of nanoparticle toxicity are associated with the production of reactive oxygen species, DNA damage, changes in the structure of proteins and cellular macromolecules, and cell membrane damage [46].

The studies included in this review carried out toxicity tests in vitro (*n* = 6), in vivo (*n* = 3), ex vivo (*n* = 1), and clinical (*n* = 1) models of annatto-containing nanostructures. In vitro tests were conducted with cell cultures of fibroblasts, keratinocytes (HaCaT), macrophages (from BALB/c mice), myoblasts (C2C12), and endothelial cells (HUVE). Significant cytotoxic effects were observed only in macrophages treated with nanocochleates containing annatto essential oil, presenting a CC_50_ value of 94.6 µg/mL, and in fibroblasts and keratinocytes treated with nanostructured lipid carriers containing 2 and 4% of annatto oil, which presented IC_50_ values of 153.64–181.93 µg/mL and 123.37–257.11 µg/mL in fibroblasts and keratinocytes, respectively [18,19].

In in vivo models, Wistar rats treated with cellulose acetate nanofibers and BALB/c mice treated with 30 mg/kg of nanocochleates containing annatto essential oil did not show clinical, biochemical, and histopathological signs of toxicity [9,19]. In the study by Carvalho et al. (2023), 1 to 10 mg/kg of nanoprecipitates containing annatto oil were injected intramuscularly in Wistar rats; only the 10 mg/kg dose showed toxic effects with associated histopathological changes in the animal’s muscles, with the appearance of areas of necrosis, infiltration of leukocytes and changes in the morphology of muscle fibers [17].

In the study by Andreo-Filho et al. (2017), solid lipid nanoparticles were topically applied to human skin at a concentration of 2 mg/cm^2^ and no changes in cell metabolism and skin cell morphology were observed. However, these biocompatibility results are limited to the small number of three participants in the study [3].

## 5. Considerations for the Nanostructuring of Annatto Products

Several factors can be considered to determine the choice of the ideal nanostructure for encapsulating annatto products. One of the main factors is the physical and chemical characteristics of the product. In the case of nonpolar products such as fixed and essential oils of annatto, lipid nanoparticles can provide a structural organization that favors greater stability, better encapsulation efficiency and better dispersion of the oil in aqueous media [47]. In the case of hydrophilic extracts, polar nanostructures, such as polymeric nanoparticles, can better encapsulate the bioactive compounds of the extract [48,49]. Among them, nanofibers have a high surface area that enhances the encapsulation efficiency of hydrophilic extracts and allows greater interaction with the biological components associated with the therapeutic effect [20]. Additionally, the choice of the ideal nanoparticle also depends on the context of the biological application, route of administration and biological targets to be achieved, which determine the physicochemical characteristics of the nanostructures that will best carry the annatto products.

The incorporation of annatto products into nanoparticles promotes the aggregation of value to these products by enhancing their biological activities and optimizing their physicochemical characteristics. These gains favor the development of effective and stable biomedical products, characteristics that are absent or incipient in the crude extract or oil [1]. Although the nanostructuring process involves the use of advanced technologies for production and characterization and consequently costs, the gains in efficacy, bioavailability and increased quality of the final product can outweigh these expenses [50]. In this sense, the experimental design for the development of pharmaceutical products must be aligned with the principles of translational research in which industrial reality scenarios are evaluated to make production scaling viable.

One of the most challenging aspects for the reproducibility and scaling of biomedical products using plant-based raw materials is the variations in chemical composition due to environmental conditions. This can make the levels of compounds responsible for the biological activities of the material inconsistent and, consequently, variations in biological response can occur [51]. The characterization and detection of the compounds responsible for the biological activities of annatto products is of great importance to promote their use in the development of pharmaceutical products. This knowledge can favor the establishment of rigorous quality control methodologies through the standardization of cultivation, extraction, and storage conditions of annatto products in order to contribute to the conservation of the compounds responsible for biological activities and the reproducibility of therapeutic activities [52].

## 6. Limitations

Limitations were observed in the development of this systematic review. The development of nanostructures with annatto extracts and oils is an approach that is still little explored in the scientific literature, making it difficult to find articles that meet the inclusion criteria. Additionally, the studies included are heterogeneous concerning the part of the plant used, extraction methodologies, and types of nanostructures developed. This scenario allows only partial comparisons between the efficacy and safety results found in different studies.

## 7. Conclusions

The main findings of the studies included in this review demonstrate that annatto extracts and oils can be used in the development of lipid, polymeric, metallic, and nanofiber nanostructures. The incorporation of annatto extracts and oils into the nanostructure optimized some physicochemical characteristics of the formulation, such as size reduction, increase in zeta potential modulus, and crystalline organization of the nanostructure. These improvements are capable of enhancing the therapeutic effects of annatto compounds, as demonstrated in the results of biological analyses in which the nanostructures showed leishmanicidal, photoprotective, antioxidant, antimicrobial, immunomodulatory efficacy, and tissue regeneration potential with no or low toxic effects in in vitro, in vivo, and clinical models. Therefore, the present work supports that the production of annatto extracts and oils nanostructures is a relevant approach to the development of new technologies for biomedical applications.

## Figures and Tables

**Table 1:** Summary of descriptive characteristics of the included studies.

Study			Intervention		Population	Outcomes
Author, Year/Country	Fruit Species/Extraction Method	Fruit Characterization Analysis	Nanoparticle Type/Production Methodology	Nanoparticle Characterization	Biological Activity Assessed/Model/Treatment Regimen	Results
Andréo-Filho et al., 2017/Brazil [3]	*Bixa orellana* oil (commercial)	Not evaluated	- Solid-Lipid Nanoparticles (SLN) Oil phase: octyl methoxycinnamate (OMC) or mineral oil (MO) or annatto oil (AO); - Low (heating) (P1) and high (high throughput and pressure homogenizer) energy process (P2);	OMC (P1): −50.41 ± 4.60 mV (ZP)/1.56 μm (PS) OMC (P2): −45.26 ± 2.23 mV (ZP)/0.20 μm (PS) OMC + AO (P1): −50.29 ± 2.67 mV (ZP)/1.42 μm (PS) OMC + AO (P2): −35.74 ± 3.31 mV (ZP)/0.20 μm (PS) MO (P2): −43.20 ± 2.16 mV (ZP)/0.20 μm (PS)	- Photoprotective efficacy (SPF) in vitro (L) with 250–450 nm wavelength range; - Skin permeation with ex vivo human abdominal skin (Franz diffusion cells); 0.01 g of OMC + AO (P1)/OMC + AO (P2)/OMC (P1)/OMC (P2); - Toxicity in 3 healthy humans (21–35 years-old); 2 mg/cm^2^ of OMC (P1)/OMC (P2)/MO (P2) spread in human’s forearms (MPT-FLIM skin images acquisition 0, 3 and 6 h after application);	↓ PS in SNLs produced by high energy process; <5% of changes in PS and <10% variation of dispersion in SNLs with 5 weeks of storage; ↑ SPF value in OMC (P2) with 27.3 ± 1.2 (*p* < 0.05); Similar SPF values in OMC (P1), OMC + AO (P1) and OMC + AO (P2) with 22.0 ± 1.0, 19.7 ± 1.5 and 21.7 ± 1.2, respectively (*p* > 0.05); No significant skin permeation of SLNs with 111, 96, 93, and 82% of recoveries from OMC (P1), OMC (P2), OMC + AO (P1) and OMC + AO (P2), respectively; No changes in cellular metabolism and morphology of human skin after 6 h SLNs application;
Carvalho et al., 2023/Brazil [17]	*Bixa orellana* oil (commercial)	72.6 ± 0.9% of δ-tocotrienol	- Polymeric *Bixa orellana* Nanodispersions (PBN); - Nanoprecipitation (solvent displacement method);	PBN: First day: 53.15 ± 0.64 nm (PS)/0.574 ± 0.032 (PdI)/18.26 ± 0.59 mV (ZP) 30th day: 59.90 ± 3.63 nm (PS)/0.574 ± 0.032 (PdI)/19.66 ± 1.45 mV (ZP)	- Toxicity in 3-week-old Wistar rats (*n* = 4); Single intramuscular injection of PNB at doses of 1, 2.5, 5 and 10 mg/kg and 4% of Tween-80 as control group (clinical parameters, biochemical and hematological analysis and muscle histopathology);	No clinical signs of PBN toxicity 4 days after injection; No muscle toxicity with 1, 2.5, and 5 mg/kg of PBN; Mild edema, hemorrhage, presence of necrotic fibers, ↑ leukocyte infiltration, and connective tissue observed in muscles with 10 mg/kg of PBN; No alterations on CPK, LDH, and myoglobin levels with 1, 2.5, and 5 mg/kg of PBN; ↑ levels of CPK, LDH, and myoglobin with 10 mg/kg of PBN (not statistically significant); No changes on erythrogram and blood lipid profile with PBN at all doses; No liver and kidney toxicity with PBN at all doses; ↑ segmented neutrophils with PBN at 2.5 and 5 mg/kg (*p* < 0.05); ↑ eosinophils with PBN at 1 mg/kg (*p* < 0.05);
Ferreira et al., 2021/Brazil [18]	*Bixa Orellana* oil (commercial)	Not evaluated	- Nanostrutured lipid carrier (NLC) loaded with 2 and 4% (*w*/*w*) of annatto oil and 10% of CP (NLCcp2 and NLCcp4) or 10% of MM (NLCmm2 and NLCmm4) - Fusion-emulsification and ultrasonication	NLCmm (2 and 4): ~170 to 190 nm (PS-DLS)/200 nm (PS-TEM)/~0.30 to 0.35 (PdI)/~−40 to −45 mV (ZP)/5.0 to 6.0 (pH)/Spherical morphology; NLCcp (2 and 4): ~170 to 190 nm (PS-DLS)/200 nm (PS-TEM)/~0.30 to 0.35 (PdI)/~−30 to −40 mV (ZP)/5.0 to 6.0 (pH)/Spherical morphology/78.92 ± 2.89% (EE NLCcp2)/50.54 ± 3.41% (EE NLCcp4);	Cytotoxicity on BALB/c 3T3 (fibroblasts) and HaCaT (keratinocytes) cells (MTT 24 h); Free annatto oil and NLCcp (2 and 4) at concentrations of 25–300 µg/mL; Leishmanicidal activity in vitro with *L. major* internalized in RAW 264.7 macrophages (resazurin cell viability assay); Free annatto oil and NLCcp (2 and 4) at concentrations of 2 and 5 µg/mL/Amphotericin B at 0.3 and 3.125 µg/mL/Glucantime at 200 and 400 µg/mL;	↑ PS of NLCcp2 after 90 days (*p* < 0.05); Phase separation of NLCmm (2 and 4) after 30 days; ↑ [ZP] on NLC with annatto oil; ↓ size variation in NLCcp4 after 90 days; NLCmm and NLCcp (2 and 4) melting points ↓ raw materials (~57 °C); Lipid crystalline nature of NLCmm and NLCcp (2 and 4) (2θ scattered angles of 7, 19, 21 and 23°); ↑ fluidity of NLCmm and NLCcp (2 and 4); No leishmanicidal activity of free annatto oil; 70–90% leishmanicidal activity of NLCcp (2 and 4); Similar leishmanicidal activity (~90%) of NLCcp 2 and 4 at 5 µg/mL (*p* > 0.05); ↑ leishmanicidal activity of NLCcp 2 and 4 (5 µg/mL) than glucantime (*p* > 0.05) No cytotoxic effect of free annatto oil on HaCaT and fibroblasts; NLCcp2 181.93 ± 8.67 µg/mL and 257.11 ± 42.11 µg/mL IC_50_ values on fibroblasts and keratinocytes, respectively; NLCcp4 153.64 ± 7.63 µg/mL and 123.37 ± 24.98 µg/mL IC_50_ values on fibroblasts and keratinocytes, respectively;
Gharpure et al., 2022/India [11]	*Bixa Orellana*/Leaves (BL), Seeds (BS) and Seed Coats (BSc) extracts/Boiling Water extraction	- Presence of flavonoids, phenolics, and glycosides on BL, BS and BSc (UV-Vis); - Aromatics and vinyl compounds on BL/Phenolics, flavonoids, aromatics and fatty acids on BS/phenolics, steroids, flavonoids, carotenoids, fatty acid, and hydrocarbons on BSc (GC-MS); - Beta-copaene and alloaromadendrene on BL/geranylgeraniol and andrographolide on BS/octadecenal, vaccenic acid and isocarpesterol on BSc (HR-MS); - Aromatics, flavonoids, steroids, alcohols, phenolics, and alkyl groups on BL, BS and BSc (NMR);	- Zinc Nanoparticles (ZnO) - Green synthesis with extracts of Leaves (L-ZnO), Seeds (S-ZnO) or Seed Coats (Sc-ZnO) of *B. orellana* follow by air-dried or calcination;	341–353 nm (L-ZnO)/378–373 nm (S-ZnO)/327–337 nm (Sc-ZnO) before and after calcination, respectively (UV-vis absorption); 467–551 nm and 470–557 nm (L-ZnO)/468–554 nm and 469–552 nm (S-ZnO)/467–554 nm and 468–558 (Sc-ZnO) before and after calcination, respectively (PL); Spherical nanocrystallites 114–344 nm (L-ZnO)/spherical and rod-like nanocrystallites 220–440 nm and 330–660 nm, respectively (S-ZnO)/spherical and rod-like nanocrystallites 257–428 nm and 428–857 nm (Sc-ZnO) (FESEM); L-ZnO spherical (169–259 nm)/Sc_ZnO spherical (278–654 nm)/S-ZnO almond-like (220–440 nm) (TEM); 37.25 nm (L-ZnO), 37.38 nm (S-ZnO), 28.65 nm (Sc-ZnO) (XRD spectra crystallite size); 81.48% (Zn) 18.52% (O) (L-ZnO)/72.31% (Zn) 27.69% (O) (S-ZnO)/80.3% (Zn) 19.7% (O) (Sc-ZnO) (EDS); 2.301 m^2^/g (L-ZnO), 2.187 m^2^/g (S-ZnO) and 2.107 m^2^/g (Sc-ZnO) (BET surface area); 5 nm pores (all ZnO) (BJH);	Anti-bacterial activity in vitro on *S. aureus*, *B. subtilis*, *E. coli*, and *P. aeruginosa*) (Well-based diffusion); L-ZnO, S-ZnO, and Sc-ZnO calcinated and uncalcinated, and free BS, BL and BSc 0.625–10 mg/mL incubated overnight; Anti-fungal activity in vitro on *Penicillium* sp., *F. oxysporum*, *A. flavus*, and *R. solani* (Well-based diffusion) L-ZnO, S-ZnO, and Sc-ZnO calcinated and uncalcinated, and free BS, BL and BSc 10 mg/mL for 4–8 days; Citotoxicity in vitro on HCT-116 cancer cell (Trypan blue staining); L-ZnO, S-ZnO, and Sc-ZnO calcinated and uncalcinated, and free BS, BL and BSc 100 µg/mL for 48 h;	No anti-bacterial activity of free BL, BS, BSc, and calcinated ZnO nanoparticles; ↑ antibacterial potential of L-ZnO uncalcinated against *S. aureus* at 10, 5, and 2.5 mg/mL and *B. subtilis* at 10 and 5 mg/mL; ↑ antibacterial potencial of S-ZnO uncalcinated against *E. coli* at 10 mg/mL; ↑ antibacterial potencial of Sc-ZnO uncalcinated against *S. aureus* at 10 mg/mL; No anti-fungal activity and cytotoxicity of all extracts and ZnO nanoparticles;
Machin et al., 2019/Cuba [19]	*Bixa orellana*/Seed essencial oil (AEO) Manual grinding with hydrodistillation	Not evaluated	- Nanocochleate loaded with AEO (NCA) prepared with purified phospholipids from soy lecithin - Dehydration-hydration method;	NCA: 52.3–96.1 nm (PS)/0.325–0.335 (PdI)/40.4 to 41.2 mV (ZP) Blank: <40 nm (PS)/0.44–0.52 (PdI)/31.1–31.3 mV (ZP)	- Anti-amastigote activity in vitro (*L. amazonensis* internalized in peritoneal macrophages from BALB/c mice) (Staining with Giemsa); - Cytotoxicity in vitro on isolated peritoneal macrophages from BALB/c mice (MTT 48 h); - Anti-leishmaniasis in vivo on female healthy BALB/c mice infected with *L. Amazonensis* (Clinical observations of body weight and deaths and cutaneous lesions measurements); Free AEO, NCA and glucantime at 30 mg/kg by intralesional route for 4 days/4 times;	↓ anti-amastigote activity of NCA (IC_50_ = 15.4 ± 1.3) compared with AEO (IC_50_ = 8.5 ± 0.8) (*p* < 0.05); ↑ cytotoxicity of free AEO (CC_50_ = 61.8 ± 5.9 µg/mL) compared with NCA (CC_50_ = 94.6 ± 2.2 µg/mL) (*p* < 0.05); Mortality and weight loss rates < 10% in vivo with free AEO and NCA; ↓ lesions cutaneous size in vivo with NCA compared with AEO (*p* < 0.05) No statistical difference on lesions cutaneous size with glucantime compare with NCA (*p* > 0.05) No successful leishmaniasis cure in vivo with free AEO and NCA;
Maitra et al., 2023/Bangladesh [10]	*Bixa orellana*/Aqueous Seed extract Heating water with sodium hydroxide	Peaks at 2930, 1615 and 1383 cm ^−1^ (FTIR).	- Silver Nanoparticles (AgNPs) - Green synthesis with *Bixa orellana* seed extract	AgNPs: 420 nm (UV-vis absorption); 2926, 1610, and 1384 cm^−1^ (FTIR peaks); 40–100 °C ~4% weight loss/100–380 °C ~21% weight loss/350–450 ~54% weight loss (Thermogravimetric analysis); Crystal plane/20–40 nm (PS) (TEM); 92.9 nm (PS)/0.310 (PdI) (DLS) 53% nano silver/21% oxygen/25% carbon (EDX);	- Antioxidant activity (DPPH scavenging); AgNPs 50, 100, and 200 µg/mL for 30 min; - Antibacterial activity on *S. aureus*, *E. coli*, *S. dysenteriae*, and *S. boydii* (Disc diffusion assay); AgNPs 15, 40, and 80 µg for 24 h; - Antitumoral activity on MCF-7 (breast cancer) (MTS); AgNPs 3.90–37.25 µg/mL for 48 h;	50.76, 62.78 and 78.86% DPPH radical scavenging of AgNPs 50, 100, and 200 µg/mL, respectively; 17 mm inhibition zone of AgNPs 80 µg dose-dependent on *S. dysenteriae*; 68.3% MCF-7 growth inhibition of AgNPs 31.25 µg/mL; 21.2% MCF-7 dose-dependent growth inhibition of AgNPs 3.9 µg/mL;
Ntohogian et al., 2018/Greece [20]	*Bixa orellana*/powder and ultrafiltrated (UF) (commercial)	Presence of bixin and norbixin on annatto and UF annatto (FTIR peaks of 3400, 2920, 2855, 1690, 1603 and 1149 cm^−1^) ↑ crystallinity of annatto and UF annatto (XRD) No differences in FTIR spectral and XRD patterns of annatto and UF annatto;	- Chitosan Nanoparticles (CS) loaded with 20, 40, and 60% (*w*/*w*) of annatto (CNA) and UF annatto (CNAF) - Ionotropic gelation (2:1 TPP-CS ratio)	Blank: 250 ± 7 nm (PS)/36 ± 3 mV (ZP) CNA: 20% = 287 ± 5 nm (PS)/34 ± 3 mV (ZP)/66.40% (EE) 40% = 310 ± 8 nm (PS)/41 ± 3 mV (ZP)/68.25% (EE) 60% = 340 ± 11 nm (PS)/46 ± 3 mV (ZP)/67.25% (EE) CNAF: 20% = 263 ± 9 nm (PS)/35 ± 3 mV (ZP)/45.01% (EE) 40% = 284 ± 10 nm (PS)/40 ± 3 mV (ZP)/62.06% (EE) 60% = 303 ± 8 nm (PS)/47 ± 3 mV (ZP)/71.54% (EE) Cream-like CNA and CNAF sunscreen emulsions (CCNA and CCNAF): 5.44 to 5.88 (pH);	Cytotoxicity on HUVE cell line (MTT 24 h); CS, Free Annatto, CNA 40% and PLA at concentrations of 100, 200, 400, 800 and 1000 mg/mL; Photoprotective efficacy (SPF) by diluted solution transmittance method; CS, CAN and CNAF 20, 40 and 60%;	No alterations on nanoparticles morphology with different annatto %; ↑ PS from 250 to 287–340 nm with ↑ annatto %; ↑ PS from 250 to 263–303 nm with ↑ UF annatto %; CNA and CNAF were amorphous by XRD analysis; ↑ CNA yield of 70.83%; ↓ CNAF yield of 46.34%; ↑ annatto % results on ↓ CNA yield; ↑ UF annatto % results on ↑ CNAF yield; Similar low cytotoxicity of CNA and CNAF on HUVE cell line compared to PLA polyester; No signs of color alteration and phase separation of CCNA and CCNAF with 90 days of storage (−4 and 25 °C); ↑ viscosity in CCNAF 20% sunscreen emulsions with 50 to 100 rmps; ↑ SPF value of CCNA (2.63–2.24) and CCNAF (2.76–2.56) compare with blank (1.00); No ↑ on SPF of nanoparticles with ↑ on annatto and UF annatto %;
Santos et al., 2021/Brazil [9]	*Bixa orellana*/seed extract/Solvent extraction (ethanol)	Presence of bixin and norbixin (UV-vis peaks of 353, 430, 455, and 482 nm and FTIR peaks of 1038, 989, and 845 cm^−1^)	- Cellulose Acetate/Annatto Nanofibers (CAAN) - Cellulose Acetate Nanofibers (CAN) - Electrospinning	CAAN: 269 ± 101 nm (PS) 0.1% (*w*/*w*) of annatto extract CAN: 468 ± 173 nm (PS)	Cytotoxicity by HET-CAM test (5 min) Cell proliferation in mouse (WT C57BL6) fibroblast primary culture cells (MTT 48 h) Biocompatibility and wound healing activity in Wistar rats (*n* = 4) 10 mm^2^ of CAAN subcutaneous inserted for 60 days	2.12 ± 0.24% of annatto global extraction yield; Endothermic peak of CAAN on 100 °C; 17.2% of CAAN degree of crystallinity; 80% of CANN mass loss at 405 °C; CAAN presented smooth and flexible mats with porous interconnections; 0 irritancy index for CAAN at all time points; No cytotoxic effect of CAAN on fibroblast cells after 48 h; ↑ attachment and spread of fibroblasts on CAAN surface after 48 h; No scarring after 15 days of CAN and CAAN implantation; Presence of residual CAAN without inflammation signs 60 days post-insertion;
Santos et al., 2023/Brazil [21]	*Bixa orellana* seed extract/Solvent extraction (ethanol)	Bixin and norbixin absorption band at 410 nm (UV-vis spectroscopy)	- Cellulose Acetate/Annatto Nanofibers (CAAN) - Cellulose Acetate Nanofibers (CAN) - Electrospinning	CAAN: 420 ± 212 nm (PS) CAN: 284 ± 130 nm (PS)	Cytotoxicity (MTT 2 and 7 days), cell morphology (SEM) and differentiation analysis (RT-qPCR) on myoblast cells (C2C12) 16 mm disk of CAN and CAAN	50 ± 3° contact angle of CAAN with water = hydrophilic property; CAAN presented smooth and homogenous porous interconnections mats; ↓ nanofiber stiffnesses after adding annatto; 98% of C2C12 cell adherence on CAAN surface (no difference compared with CAN); Similar C2C12 cell viability on CAN and CAAN after 2 days; ↑ C2C12 cell viability on CAAN compared with CAN after 7 days; ↑ C2C12 cell viability on CAAN surface compared with CAN after 2 days; Aligned and elongated C2C12 cell morphology on CAN surface; Thinner and randomly distributed C2C12 cell morphology on CAAN surface; ↑ C2C12 myogenic markers (*Myf5*, *MyoD*, *MyoG*, and *Desmin*) gene expression on CAAN after 7 days compared with CAN; ↓ C2C12 myogenic markers *Myf5*, *MyoD*, *Desmin*, and ↑ *MyoG* gene expression on CAAN after 14 days compared with CAN;

Legend: BJH = Teller, Brunauer, Emmett, Barrett, Joyner, Halenda; CC_50_ = 50% cytotoxic concentration; CP = cetyl palmitate; CPK = creatine kinase; DLS = Dynamic Light Scattering; DPPH = 2,2-Diphenyl-1-picrylhydrazyl; EDS = field emission scanning electron microscopy; EDX = Energy-dispersive X-ray spectroscopy; EE = Entrapment Efficiency; FESEM = Field Emission Scanning Electron Microscopy; FTIR = Fourier Transformation Infrared Spectroscopy; GC-MS = Gas Chromatography–Mass Spectrometry; HR-MS = High Resolution Mass Spectrometry; IC_50_ = half maximal inhibitory concentrations; LDH = Lactate dehydrogenase; MM = myristyl myristate; MPT-FLIM = Multiphoton Tomography with Fluorescence Lifetime Imaging Microscopy; MTS = 3-(4,5-dimethylthiazol-2-yl)-5-(3-carboxymethoxyphenyl)-2-(4-sulfophenyl)-2H-tetrazolium; MTT = 3-(4,5-dimethylthiazol-2-yl)-2,5-diphenyl tetrazolium bromide; NMR = Nuclear Magnetic Resonance spectroscopy; PdI = Polydispersivity index; PL = Photoluminescence; PLA = poly(L-lactide); PS = particle size; OMC = octyl methoxycinnamate; TEM = Transmission Electron Microscopy; TPP = Tripolyphosphate; UV-vis = Ultraviolet–visible spectroscopy; XRD = X-ray diffractometry; ZP = zeta potential; ↓ = Decrease; ↑ = Increase.

**Table 2:** Results of the quality analysis of the included studies.

Quality Analysis																				
Author	Year	Experimental Design/Scientific Report														Results				Ethical Statement
		1	2	3	4	5	6	7	8	9	10	11	12	13	14	15	16	17	18	19
Andréo-Filho et al. [3]	2017	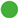	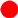	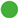	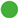	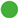	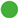	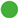	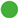	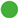	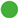	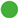	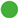	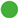	-	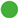	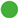	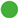	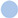	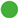
Carvalho et al. [17]	2023	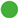	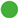	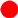	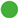	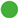	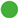	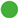	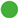	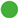	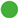	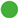	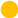	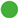	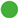	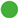	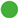	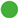	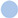	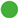
Ferreira et al [18]	2021	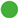	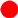	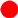	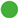	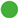	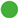	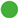	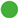	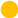	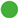	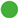	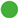	-	-	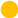	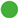	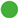	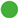	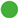
Gharpure et al. [11]	2023	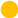	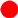	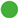	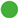	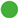	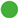	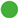	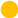	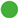	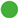	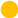	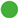	-	-	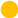	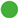	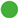	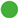	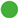
Machin et al. [19]	2019	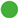	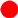	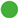	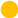	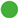	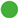	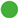	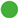	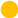	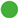	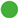	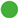	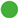	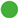	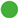	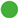	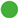	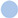	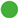
Maitra et al. [10]	2023	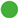	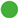	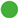	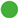	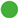	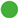	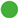	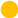	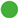	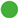	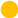	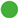	-	-	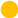	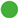	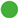	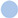	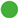
Ntohogian et al. [20]	2018	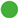	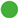	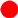	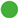	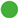	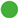	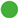	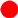	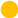	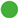	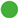	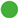	-	-	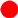	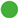	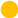	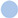	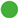
Santos et al. [9]	2021	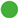	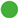	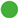	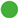	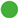	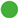	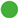	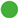	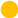	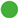	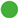	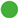	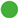	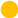	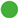	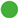	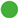	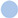	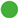
Santos et al. [21]	2023	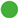	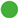	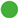	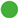	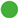	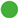	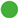	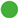	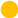	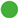	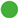	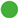	-	-	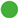	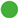	-	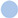	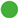

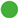
 High quality, 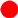 Low quality, 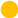 Some concerns, 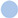 No information, - Not applicable.

**Table 3:** Results of the risk of bias using Syrcle’s risk of bias of the in vivo included studies.

Syrcle’s Risk of Bias											
Author	Year	Questions									
		1	2	3	4	5	6	7	8	9	10
Carvalho et al. [17]	2023	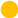	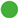	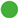	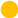	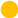	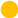	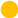	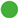	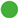	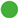
Maitra et al. [10]	2023	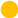	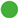	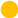	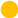	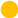	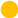	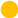	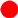	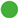	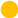
Santos et al. [9]	2021	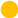	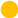	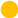	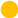	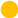	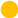	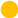	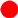	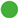	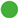

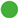
 Low risk, 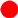 High risk, 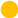 Unclear.

## Supplementary Materials

The following supporting information can be downloaded at: https://www.mdpi.com/article/10.3390/pharmaceutics16101275/s1, Prisma 2020 checklist, Table S1: Search results; Table S2: Exclusion criteria; Table S3: Quality analysis questions; Table S4: Syrcle’s risk of bias questions. References [53,54,55,56,57,58,59,60,61,62,63,64] are cited in the Supplementary Materials.
