# Supplementary material for: Annatto (Bixa orellana)-Based Nanostructures for Biomedical Applications—A Systematic Review
Source: Pharmaceutics. 2024 Sep 29;16(10):1275. doi: 10.3390/pharmaceutics16101275 (PMC11510392; doi:10.3390/pharmaceutics16101275)
Supplement: Supplementary file 1 [file pharmaceutics-16-01275-s001.zip › pharmaceutics-3127249-supplementary.pdf]

PRISMA 2020 Checklist

Section and Topic	Item #	Checklist item	Location where item is reported (page number)
**TITLE**			
Title	1	Identify the report as a systematic review.	1
**ABSTRACT**			
Abstract	2	See the PRISMA 2020 for Abstracts checklist.	1
**INTRODUCTION**			
Rationale	3	Describe the rationale for the review in the context of existing knowledge.	2 and 3 (topic 1)
Objectives	4	Provide an explicit statement of the objective(s) or question(s) the review addresses.	3 (topic 1)
**METHODS**			
Eligibility criteria	5	Specify the inclusion and exclusion criteria for the review and how studies were grouped for the syntheses.	3 (topic 2.2)
Information sources	6	Specify all databases, registers, websites, organisations, reference lists and other sources searched or consulted to identify studies. Specify the date when each source was last searched or consulted.	3 (topic 2.3)
Search strategy	7	Present the full search strategies for all databases, registers and websites, including any filters and limits used.	Supplementary table 1
Selection process	8	Specify the methods used to decide whether a study met the inclusion criteria of the review, including how many reviewers screened each record and each report retrieved, whether they worked independently, and if applicable, details of automation tools used in the process.	4 (topic 2.4)
Data collection process	9	Specify the methods used to collect data from reports, including how many reviewers collected data from each report, whether they worked independently, any processes for obtaining or confirming data from study investigators, and if applicable, details of automation tools used in the process.	4 (topic 2.4)
Data items	10a	List and define all outcomes for which data were sought. Specify whether all results that were compatible with each outcome domain in each study were sought (e.g. for all measures, time points, analyses), and if not, the methods used to decide which results to collect.	4 (topic 2.4)
	10b	List and define all other variables for which data were sought (e.g. participant and intervention characteristics, funding sources). Describe any assumptions made about any missing or unclear information.	4 (topic 2.4)
Study risk of bias assessment	11	Specify the methods used to assess risk of bias in the included studies, including details of the tool(s) used, how many reviewers assessed each study and whether they worked independently, and if applicable, details of automation tools used in the process.	4 (topic 2.5)
Effect measures	12	Specify for each outcome the effect measure(s) (e.g. risk ratio, mean difference) used in the synthesis or presentation of results.	4 (topic 2.4)
Synthesis methods	13a	Describe the processes used to decide which studies were eligible for each synthesis (e.g. tabulating the study intervention characteristics and comparing against the planned groups for each synthesis (item #5)).	3 (topic 2.2)
	13b	Describe any methods required to prepare the data for presentation or synthesis, such as handling of missing summary statistics, or data conversions.	4 (topic 2.4)
	13c	Describe any methods used to tabulate or visually display results of individual studies and syntheses.	4 (topic 2.4)
	13d	Describe any methods used to synthesize results and provide a rationale for the choice(s). If meta-analysis was performed, describe the model(s), method(s) to identify the presence and extent of statistical heterogeneity, and software package(s) used.	4 (topics 2.4 and 2.5)
	13e	Describe any methods used to explore possible causes of heterogeneity among study results (e.g. subgroup analysis, meta-regression).	Not applied

PRISMA 2020 Checklist

Section and Topic	Item #	Checklist item	Location where item is reported (page number)
	13f	Describe any sensitivity analyses conducted to assess robustness of the synthesized results.	Not applied
Reporting bias assessment	14	Describe any methods used to assess risk of bias due to missing results in a synthesis (arising from reporting biases).	4 (topic 2.5)
Certainty assessment	15	Describe any methods used to assess certainty (or confidence) in the body of evidence for an outcome.	Not applied
**RESULTS**			
Study selection	16a	Describe the results of the search and selection process, from the number of records identified in the search to the number of studies included in the review, ideally using a flow diagram.	7 (topic 3.1)
	16b	Cite studies that might appear to meet the inclusion criteria, but which were excluded, and explain why they were excluded.	Supplementary table 2
Study characteristics	17	Cite each included study and present its characteristics.	7 and 8 (topic 3.2)
Risk of bias in studies	18	Present assessments of risk of bias for each included study.	8 (topic 3.3) and 13 (tables 2 and 3)
Results of individual studies	19	For all outcomes, present, for each study: (a) summary statistics for each group (where appropriate) and (b) an effect estimate and its precision (e.g. confidence/credible interval), ideally using structured tables or plots.	9 to 12 (table 1)
Results of syntheses	20a	For each synthesis, briefly summarise the characteristics and risk of bias among contributing studies.	8 (topic 3.3) and 13 and 14 (topic 3.4)
	20b	Present results of all statistical syntheses conducted. If meta-analysis was done, present for each the summary estimate and its precision (e.g. confidence/credible interval) and measures of statistical heterogeneity. If comparing groups, describe the direction of the effect.	Not applied
	20c	Present results of all investigations of possible causes of heterogeneity among study results.	13 and 14 (topic 3.4)
	20d	Present results of all sensitivity analyses conducted to assess the robustness of the synthesized results.	Not applied
Reporting biases	21	Present assessments of risk of bias due to missing results (arising from reporting biases) for each synthesis assessed.	8 (topic 3.3) and 13 (tables 2 and 3)
Certainty of evidence	22	Present assessments of certainty (or confidence) in the body of evidence for each outcome assessed.	Not applied
**DISCUSSION**			
Discussion	23a	Provide a general interpretation of the results in the context of other evidence.	14 to 20 (topic 4)
	23b	Discuss any limitations of the evidence included in the review.	20 and 21 (topic 5)

PRISMA 2020 Checklist

Section and Topic	Item #	Checklist item	Location where item is reported (page number)
	23c	Discuss any limitations of the review processes used.	20 and 21 (topic 5)
	23d	Discuss implications of the results for practice, policy, and future research.	20 (topic 6)
**OTHER INFORMATION**			
Registration and protocol	24a	Provide registration information for the review, including register name and registration number, or state that the review was not registered.	3 (topic 2.1)
	24b	Indicate where the review protocol can be accessed, or state that a protocol was not prepared.	3 (topic 2.1)
	24c	Describe and explain any amendments to information provided at registration or in the protocol.	Not applied
Support	25	Describe sources of financial or non-financial support for the review, and the role of the funders or sponsors in the review.	21
Competing interests	26	Declare any competing interests of review authors.	21
Availability of data, code and other materials	27	Report which of the following are publicly available and where they can be found: template data collection forms; data extracted from included studies; data used for all analyses; analytic code; any other materials used in the review.	3 and 4

From: Page MJ, McKenzie JE, Bossuyt PM, Boutron I, Hoffmann TC, Mulrow CD, et al. The PRISMA 2020 statement: an updated guideline for reporting systematic reviews. BMJ 2021;372:n71. doi: 10.1136/bmj.n71

PRISMA 2020 Checklist

Table S1

Data base	Search date	String	Results
Pubmed	06/06/2023	("Bixaceae"[Mesh] OR "annatto" [Supplementary Concept] OR "Bixa orellana" [Supplementary Concept] OR urucum) AND (Nano OR "Nanotechnology"[Mesh] OR "Nanoparticle Drug Delivery System"[Mesh] OR "Theranostic Nanomedicine"[Mesh] OR "Nanoparticles"[Mesh] OR "Nanostructures"[Mesh] OR "Nanogels"[Mesh] OR "Nanoconjugates"[Mesh] OR "Nanocapsules"[Mesh])	24
Medline	05/06/2023	(("Bixa orellana" OR annatto OR Urucum) AND (Nano* OR Nanotechnology* OR nanomaterials* OR Nanobiotechnology* OR Nanomedicine* OR Nanoparticles* OR Nanogels* OR Nanostructures*))	35
LILACS	05/06/2023	(("Bixa orellana" OR annatto OR Urucum) AND (Nano* OR Nanotechnology* OR nanomaterials* OR Nanobiotechnology* OR Nanomedicine* OR Nanoparticles* OR Nanogels* OR Nanostructures*))	2
FSTA	05/06/2023	("Bixa orellana" OR annatto OR Urucum) AND (Nano* OR Nanotechnology* OR nanomaterials* OR Nanobiotechnology* OR Nanomedicine* OR Nanoparticles* OR Nanogels* OR Nanostructures*) Search field "AB resume" applied.	8
CINAHL	05/06/2023	("Bixa orellana" OR annatto OR Urucum) AND (Nano* OR Nanotechnology* OR nanomaterials* OR Nanobiotechnology* OR	10

		Nanomedicine* OR Nanoparticles* OR Nanogels* OR Nanostructures*)	
Scopus	05/06/2023	TITLE-ABS-KEY-AUTH (("Bixa orellana" OR annatto OR urucum) AND (nano* OR nanotechnology* OR nanomaterials* OR nanobiotechnology* OR nanomedicine* OR nanoparticles* OR nanogels* OR nanostructures*))	44
Web of Science	05/06/2023	ALL=((("Bixa orellana" OR annatto OR urucum) AND (nano* OR nanotechnology* OR nanomaterials* OR nanobiotechnology* OR nanomedicine* OR nanoparticles* OR nanogels* OR nanostructures*)))	57
Science Direct	05/06/2023	(("Bixa orellana" OR annatto OR urucum) AND (nano$ OR nanotechnology OR nanomaterial$ OR nanomedicine OR nanoparticle$ OR nanostructure$))) Filter research article applied	143
Embase	05/06/2023	(('Bixa orellana'/exp OR annatto OR urucum) AND ('nanomedicine'/exp OR 'nanoparticle'/exp OR 'nanomaterial'/exp OR nano OR 'lipid nanoparticle'/exp))	20
ProQuest	05/06/2023	(("Bixa orellana" OR annatto OR Urucum) AND (Nano* OR Nanotechnology* OR nanomaterials* OR nanoparticles* OR nanomedicine OR nanostructures))	365

Table S2. Exclusion Criteria.

Author/ year	Criteria of exclusion
Barbosa et al., 2022 [1]	4
Bittencourt et al., 2018 [2]	4
Duran et al., 2021 [3]	4
Huáman et al., 2021 [4]	2
Huáman Aguirre et al., 2021 [5]	2
Kumar et al., 2021 [6]	2
Maurya et al., 2019 [7]	4
Pal et al., 2016 [8]	4
Prada et al., 2016 [9]	4
Oliveira et al., 2022 [10]	4
Silva et al., 2015 [11]	1

Criterias of exclusion:

1- Non-nanostructured *Bixa orellana* extract;

2- Nanostructured compounds isolated from *Bixa orellana;*

3- Lack of evaluation of morphology, hydrodynamic diameter, zeta potential or polydispersion index of *Bixa orellana* nanostructures;

4- Biological activity or toxicity of *Bixa orellana* nanostructures not evaluated;

5- Review articles, book chapters, theses, letters, personal opinions, conference abstracts, and patentes;

Table S3. **Quality analysis questions**

1	Is the origin of annatto clearly described?
2	Was the characterization of annatto extract or oil performed?
3	Is there a description of the extraction methodology of annatto extract or oil?
4	Does the study clearly describe the production/synthesis methodologies and characterization analysis of the nanostructures? Are the names of the equipment used described?
5	Is the study abstract clear and does it contain all relevant information? As objectives, experimental design, key methods used and relevant results?
6	Does the introduction bring information that contextualizes and justifies the study?
7	Is the research question clearly stated in the study objectives?
8	Have the details of the statistical analysis used in each analysis been reported?
9	Are the steps of the experimental procedures and their intervals and measurements described clearly and with a level of detail that allows for replication?
10	Has all relevant information about the characteristics of the models used been clearly described?
11	Are control groups present?
12	Were the biological variables considered for measuring the outcomes clearly described?
13	Is there a description of approval by an ethics committee and has the name of the committee been reported?
14	Were the experimental conditions of animal allocation and supplementation clearly reported?
15	Do the results contain data from all experiments clearly described with an indication of the statistically significant difference value (p value)?
16	Is the interpretation of the results contextualized with the objectives of the study? And were the limitations of the study described?
17	Is there a description of the results that indicates the possibility or not of translation for future experiments in other experimental models?

18	Did the study provide raw data regarding the results? (These data refer to the complete data used in the statistics and can be made available on some platform and the link must be present in the article)
19	Is there a declaration of the presence or absence of conflicts of interest in the study?

Table S4. **Syrcle's risk of bias questions**

1	Was the allocation sequence adequately generated and applied?
2	Were the groups similar at baseline or were they adjusted for confounders in the analysis?
3	Was the allocation to the different groups adequately concealed during?
4	Were the animals randomly housed during the experiment?
5	Were the caregivers and/or investigators blinded from knowledge which intervention each animal received during the experiment?
6	Were animals selected at random for outcome assessment?
7	Was the outcome assessor blinded?
8	Were incomplete outcome data adequately addressed?
9	Are reports of the study free of selective outcome reporting?
10	Was the study apparently free of other problems that could result in high risk of bias?

References

1. Barbosa R.M., Leite A.M., García-Villén F., et al. Hybrid Lipid/Clay Carrier Systems Containing Annatto Oil for Topical Formulations. *Pharmaceutics*. 2022, 14(5):1067. Published 2022 May 17. doi:10.3390/pharmaceutics14051067
2. Bitencourt A.P.R., Duarte J.L., Oliveira A.E.M.F.M., et al. Preparation of aqueous nanodispersions with annatto (Bixa orellana L.) extract using an organic solvent-free and low energy method. *Food Chem*. 2018, 257:196-205. doi:10.1016/j.foodchem.2018.02.067
3. Naranjo-Durán, A.M., Quintero-Quiroz, J., Rojas-Camargo, J. *et al.* Modified-release of encapsulated bioactive compounds from annatto seeds produced by optimized ionic gelation techniques. *Sci Rep*. 2021, 11, 1317. https://doi.org/10.1038/s41598-020-80119-1
4. Huamán A.A., Celestino M.R., Quintana M.E.. Theoretical and experimental study of solar cells based on nanostructured films of TiO2 sensitized with natural dyes extracted from *Zea mays* and *Bixa orellana*. *RSC Adv*. 2021, 11(16):9086-9097. Published 2021 Mar 1. doi:10.1039/d1ra01043c
5. Huamán Aguirre, A. A., Salazar Salinas, K., & Quintana Cáceda, M. Molecular interaction of natural dye based on Zea mays and Bixa orellana with nanocrystalline TiO2 in dye sensitized solar cells. *Journal of Electrochemical Science and Engineering*, 2021, *11*(3), 179–195. https://doi.org/10.5599/jese.1014
6. Kumar, A., Bera, S., Singh, M. *et al.* Molecular Interactions of Silica Nanoparticles and Biomolecule-Functionalized Silica Nanoparticles with *Bixa orellana* L. Plant DNA. *Silicon.* 2022*,* **14**, 1407–1419. https://doi.org/10.1007/s12633-020-00913-4
7. Maurya I. C., Singh S., Senapati S., Srivastava P., Bahadur L. Green synthesis of TiO2 nanoparticles using Bixa orellana seed extract and its application for solar cells. *Solar Energy*. 2019, 194. 952–958. 10.1016/j.solener.2019.10.090.
8. Pal S., Malhotra S., Naik S. Development of cosmeceutical products from nano-sized active colour constituents of Ratanjot, Seabuckthorn and Annatto. *Int J Pharm Pharm Sci.* 2016, 8. 232-237.

9. Prada A.L., Bitencourt A.P., Amado J.R., Cruz R.A., Carvalho J.C., Fernandes C.P. Development and Characterization of Cassia grandis and Bixa orellana Nanoformulations. *Curr Top Med Chem*. 2016, 16(18):2057-2065. doi:10.2174/1568026616666160215161103
10. Oliveira S.D.S.D.C., Sarmento E.D.S., Marinho V.H., Pereira R.R., Fonseca L.P., Ferreira I.M. Green Extraction of Annatto Seed Oily Extract and Its Use as a Pharmaceutical Material for the Production of Lipid Nanoparticles. *Molecules*. 2022, 27(16):5187. doi:10.3390/molecules27165187
11. Silva E.K., Meireles M.A. Influence of the degree of inulin polymerization on the ultrasound-assisted encapsulation of annatto seed oil. *Carbohydr Polym*. 2015, 133:578-586. doi:10.1016/j.carbpol.2015.07.025
